# Telehealth and In-Person Behavioral Health Services in Rural Communities Before and During the COVID-19 Pandemic: Multisite Prospective Cohort Study

**DOI:** 10.2196/47047

**Published:** 2023-09-18

**Authors:** Marcia M Ward, Fred Ullrich, Divya Bhagianadh, Eve-Lynn Nelson, James P Marcin, Knute D Carter, Kari Beth Law, Carly McCord, Jonathan Neufeld, Kimberly A S Merchant

**Affiliations:** 1 Department of Health Management and Policy University of Iowa Iowa City, IA United States; 2 School of Social Work Rutgers University New Brunswick, NJ United States; 3 Department of Pediatrics Kansas University Medical Center Kansas City, KS United States; 4 Department of Pediatrics University of California Davis School of Medicine Sacramento, CA United States; 5 Department of Biostatistics University of Iowa Iowa City, IA United States; 6 Department of Behavioral Medicine and Psychiatry West Virginia University Morgantown, WV United States; 7 Department of Psychiatry and Behavioral Sciences and Educational Psychology Texas A&M University College Station, TX United States; 8 Institute for Health Informatics University of Minnesota Minneapolis, MN United States

**Keywords:** anxiety, behavior, behavioral health, COVID-19, depression, digital health, eHealth, mental health, mHealth, pandemic, rural health services, rural, telehealth, telemedicine

## Abstract

**Background:**

The COVID-19 pandemic triggered widespread adjustments across the US health care system. Telehealth use showed a substantial increase in mental health conditions and services due to acute public health emergency (PHE) behavioral health needs on top of long-standing gaps in access to behavioral health services. How health systems that were already providing behavioral telehealth services adjusted services and staffing during this period has not been well documented, particularly in rural areas with chronic shortages of behavioral health providers and services.

**Objective:**

This study investigates patient and treatment characteristic changes from before the COVID-19 PHE to during the PHE within both telehealth and in-person behavioral health services provided in 95 rural communities across the United States.

**Methods:**

We used a nonrandomized, prospective, multisite research design involving 2 active treatment groups. The telehealth cohort included all patients who initiated telehealth treatment regimens during the data collection period. A comparison group included a cohort of patients who initiated in-person treatment regimen. Patient enrollment occurred on a rolling basis, and data collection was extended for 3 months after treatment initiation for each patient. Chi-square tests compared changes from pre-PHE to PHE time periods within telehealth and in-person treatment cohorts. The dependent measures included patient diagnosis, clinicians providing treatment services, and type of treatment services provided at each encounter. The 4780 patients in the telehealth cohort and the 6457 patients in the in-person cohort had an average of 3.5 encounters during the 3-month follow-up period.

**Results:**

The encounters involving anxiety, dissociative, and stress-related disorders in the telehealth cohort increased from 30% (698/2352) in the pre-PHE period to 35% (4632/12,853) in the PHE period (*P*<.001), and encounters involving substance use disorders in the in-person cohort increased from 11% (468/4249) in the pre-PHE period to 18% (3048/17,047) in the PHE period (*P*<.001). The encounters involving treatment service codes for alcohol, drug, and medication-assisted therapy in the telehealth cohort increased from 1% (22/2352) in the pre-PHE period to 11% (1470/13,387) in the PHE period (*P*<.001); likewise, encounters for this type of service in the in-person cohort increased from 0% (0/4249) in the pre-PHE period to 16% (2687/17,047) in the PHE period (*P*<.001). From the pre-PHE to the PHE period, encounters involving 60-minute psychotherapy in the telehealth cohort increased from 8% (190/2352) to 14% (1802/13,387; *P*<.001), while encounters involving group therapy in the in-person cohort decreased from 12% (502/4249) to 4% (739/17,047; *P*<.001).

**Conclusions:**

The COVID-19 pandemic challenged health service providers, and they adjusted the way both telehealth and in-person behavioral therapy services were delivered. Looking forward, future research is needed to explicate the interaction of patient, provider, setting, and intervention factors that influenced the patterns observed as a result of the COVID-19 pandemic.

## Introduction

The COVID-19 pandemic triggered widespread adjustments across the US health care system. Once the public health emergency (PHE) was declared, several major legislative initiatives and policy waivers were enacted to expand web-based access to needed health care [1]. The PHE and associated policy changes prompted providers and patients to consider telehealth as a specific treatment option to address the need for social distancing and related reasons [2]. Many providers adopted telehealth for the first time or expanded their telehealth offerings [3-6].

As the COVID-19 pandemic unfolded, behavioral health concerns rose rapidly [7-9]. The largest increase in telehealth use was for mental health conditions and services due to rising acute behavioral health needs on top of longstanding gaps in access to behavioral health services [4,5,7,10-12]. Since the onset of the COVID-19 pandemic, publications have appeared describing how health systems implemented behavioral telehealth to meet this new demand [13]. In particular, multiple publications highlighted the challenges to ramping up behavioral telehealth services and the perceptions among both patients and providers toward their adoption [4,5,7,10-12,14] but the literature on the pandemic lacks information on the changes in existing behavioral health services regarding adjustments to provider type and services to meet the increased demand and changing patient needs triggered by the pandemic, particularly in rural areas with chronic shortages of behavioral health providers and services [15]. Previous population studies reported increases in certain psychiatric disorders, such as anxiety and substance use, during the COVID-19 pandemic [16-19]. We hypothesize that if those findings are replicated in this study, then the treatment types used by behavioral health providers would have adjusted accordingly to meet the needs of the changing patient population. In addition, particularly lacking are studies that examine changes in both telehealth and in-person behavioral health services and those serving rural populations. Thus, the purpose of this analysis is to compare the profile of telehealth and in-person treatments across a large national sample of rural behavioral health care providers during the time period immediately before the PHE with those during the early phases of the PHE.

## Methods

### Overview

This study pools data from 2 grant programs funded by the Office for the Advancement of Telehealth in the Health Resources and Services Administration of the US Department of Health and Human Services: the Substance Abuse Treatment Telehealth Network Grant Program funded from September 2017 to August 2020 and the Evidence-Based Tele-Behavioral Health Network Program funded from September 2018 to August 2021. Through these 2 programs, 17 grantees provided telebehavioral health services to 95 rural communities in 13 states (California, Indiana, Kansas, Kentucky, Maryland, Massachusetts, Minnesota, Missouri, Oregon, Pennsylvania, South Dakota, Texas, and West Virginia). For both grant programs, “rural” was defined by Health Resources and Services Administration as all nonmetro counties, all metro census tracts with rural-urban commuting area (RUCA) codes 4-10, and large area metro census tracts of at least 400 square miles in area with population densities of 35 or less per square mile with RUCA codes 2-3. The originating sites included behavioral health clinics, substance use treatment facilities, primary care outpatient clinics, rural health clinics, federally qualified health clinics, critical access hospitals, and schools; for telehealth, the sites providing services included these and larger medical centers. The grantees all had established telehealth networks before grant funding that delivered behavioral telehealth services through synchronous video connections. According to funding guidance, the implemented behavioral health services followed standard therapeutic guidelines tailored to the needs, resources, and capacities of the grantees and their rural communities.

This study was a multisite, nonrandomized, prospective research design that involved 2 active usual-care treatment cohorts. The telehealth cohort included all patients who began telehealth-based treatment as part of either grant program during the data collection periods. Grantees were asked to provide data on patients who began in-person treatment, had similar primary complaints or diagnoses, demographics, and received comparable in-person treatment to their telehealth cohort. This group of patients forms the in-person cohort. Patients were enrolled on a rolling basis. The data collection at each of the sites focused on initial visits and all encounters that followed in the first 3 months of treatment for each patient.

The Rural Telehealth Research Center was funded by the Office for the Advancement of Telehealth to serve as a data coordinating center for the programs. We identified 26 component data elements [20], including patient and treatment characteristics, and created a data element dictionary, study protocol, and Excel (Microsoft Corp)-based tool for data collection.

### Data and Analysis

Grantees transmitted data to the coordinating center quarterly. Overall, 2 time periods relevant to the COVID-19 PHE were defined. Data transmitted from October 2019 to March 2020 (5 months) were considered in the pre-PHE time period, and data transmitted from April 2020 to July 2021 (15 months) were considered in the PHE time period. Patient and treatment characteristics during each encounter were compared across the 2 time periods with chi-square tests using Stata 16 within the telehealth cohort and the in-person cohort. The dependent measures were collected at each encounter and included patient diagnosis, clinician type providing treatment services, and type of treatment services provided. Patient diagnosis was recorded as International Classification of Disease-10 codes; clinician providing treatment were recorded from a checklist or open text field; and treatment service type was recorded in the form of current procedural terminology (CPT) or Health Common Procedure Coding System (HCPCS) codes.

### Ethical Considerations

Given that no experimental interventions were involved and all data were deidentified before transfer to the data coordinating center, the study protocol for data analysis was deemed not human subjects research by the University of Iowa’s institutional review board (IRB) chair (IRB-01 #201912016). The procedures followed were in accordance with the ethical standards of the responsible committee on human experimentation (institutional and national) and with the Helsinki Declaration of 1975, as revised in 2000. Study protocols at each contributing grantee were reviewed by that grantee’s IRB.

Data transfer and use agreements between each grantee and the University of Iowa were established to protect the confidentiality of the data being analyzed. We performed data monitoring and management activities to verify data accuracy, completeness, consistency, and timeliness. Office of Management and Budget clearance was received in October 2019, and grantees provided data from then until July 2021.

## Results

Grantees provided data on both the telehealth and in-person cohorts using the data collection tool, resulting in a data set with 4780 patients in the telehealth cohort and 6457 patients in the in-person cohort. During the first 3 months of treatment, individuals in the telehealth cohort averaged 3.5 encounters and those in the in-person cohort averaged 3.4 encounters. The number of encounters did not differ significantly between time periods for either the telehealth or in-person cohorts (*P*=.63 and *P*=.58, respectively). Differences within the telehealth and in-person cohorts across the 2 time periods with respect to patient demographic characteristics are presented in Multimedia Appendix 1.

As shown in Table 1, the patient diagnosis recorded at each encounter differed within each treatment cohort between time periods. Within the telehealth cohort, there was an increase in the percent of encounters involving anxiety, dissociative, and stress-related disorders from 30% (698/2352) in the pre-PHE period to 35% (4632/12,853) in the PHE period (*P*<.001), while the percent of encounters involving non–mental health diagnoses decreased from 15% (353/2352) in the pre-PHE period to 6% (763/12,853) in the PHE period (*P*<.001). Within the in-person cohort, the percent of encounters involving substance use disorders increased from 11% (468/4249) in the pre-PHE period to 18% (3048/17,047) in the PHE period (*P*<.001), while the percent of encounters involving mood (affective) disorders decreased from 37% (1550/4249) in the pre-PHE period to 28% (4685/17,047) in the PHE period (*P*<.001).

As shown in Table 2, the treatment service type for encounters in the form of CPT or HCPCS codes differed significantly within the 2 treatment cohorts between time periods. Within the telehealth cohort, from the pre-PHE period to the PHE period, the percent of encounters involving HCPCS codes for alcohol, drug, and medication-assisted therapy increased substantially from 1% (22/2352) to 11% (1470/13,387; *P*<.001) as did the percent of encounters involving 60-minute psychotherapy, which increased from 8% (190/2352) to 14% (1802/13,387; *P*<.001), while the percent of encounters involving health and behavior interviews decreased from 8% (197/2352) to 0.2% (25/13,387; *P*<.001). Within the in-person cohort, from the pre-PHE period to the PHE period, the percent of encounters involving HCPCS codes for alcohol, drug, and medication-assisted therapy increased substantially from 0% (0/4249) to 16% (2687/17,047; *P*<.001) while the percent of encounters involving group therapy decreased from 12% (502/4249) to 4% (739/17,047; *P*<.001).

As shown in Table 3, the clinician type for encounters significantly differed between time periods within the 2 treatment cohorts. From the pre-PHE period to the PHE period, within the telehealth cohort, encounters involving licensed or provisionally licensed counselors increased from 22% (508/2353) to 27% (3674/13,387; *P*<.001) and from 12% (293/2353) to 15% (2040/13,387; *P*<.001) for clinical psychologists, while the percent of encounters involving clinical social workers decreased from 32% (783/2352) to 22% (2913/13,387; *P*<.001) and from 18% (416/2352) to 9% (1209/13,387; *P*<.001) for psychiatric and mental health advanced practice providers. From the pre-PHE period to the PHE period within the in-person cohort, encounters involving licensed or provisionally licensed professional counselors increased from 0% (0/4249) to 11% (1881/17,047; *P*<.001), from 2% (88/4249) to 11% (1797/17,047; *P*<.001) for clinical psychologists, and from 7% (299/4249) to 9% (1477/17,047; *P*<.001) for psychiatrists and other physicians. At the same time, the percent of encounters involving clinical social workers decreased substantially from 86% (3649/4249) to 55% (9313/17,047; *P*<.001) from the pre-PHE period to the PHE period. In both cohorts, the percent of unknown clinician type was almost 0% (16/6601) in the pre-PHE period but exceeded 10% (3215/30,434) in the PHE period (*P*<.001).

**Table 1:** Frequencies and percentages of encounters with each diagnostic category for the 2 treatment cohorts by time period. Categories with low n (ie, less than 3%) have been combined in to “Other mental health primary diagnoses.”

Patient diagnostic category	Telehealth cohort			In-person cohort	
	Pre-PHE^a^, n (%)	PHE, n (%)	Pre-PHE, n (%)		PHE, n (%)
Anxiety, dissociative, stress- related, somatoform, and other nonpsychotic mental disorders (F40-F48)	698 (29.7)	4632 (34.6)	1762 (41.5)		6936 (40.7)
Mood (affective) disorders (F30-F39)	686 (29.2)	4028 (30.1)	1550 (36.5)		4685 (27.5)
Mental and behavioral disorders due to psychoactive substance use (F10-F19)	405 (17.2)	2374 (17.7)	468 (11)		3048 (17.9)
Behavioral and emotional disorders with onset usually occurring in childhood and adolescence (F90-F98)	152 (6.5)	941 (7)	152 (3.7)		941 (5.5)
Other mental health primary diagnoses	58 (2.5)	115 (4.8)	250 (5.9)		720 (4.2)
Nonmental health primary diagnosis	353 (15)	763 (5.7)	67 (1.6)		717 (4.2)

^a^PHE: public health emergency.

**Table 2:** Frequencies and percentages of encounters with each treatment service type for the 2 treatment cohorts by time period. Categories with low n (ie, less than 2%) have been combined into the “Other” category.

Treatment service type	Telehealth cohort		In-person cohort	
	Pre-PHE^a^, n (%)	PHE, n (%)	Pre-PHE, n (%)	PHE, n (%)
90832 Individual psychotherapy (30 min)	233 (9.9)	1478 (11)	221 (5.2)	1562 (9.2)
90834 Individual psychotherapy (45 min)	478 (20.3)	2371 (17.7)	1055 (24.8)	2676 (15.7)
90837 Individual psychotherapy (60 min)	190 (8.1)	1802 (13.5)	1273 (30)	4788 (28.1)
90853 Group psychotherapy (60 min)	129 (5.5)	720 (5.4)	502 (11.8)	739 (4.3)
90791-90792 Psychiatric diagnostic evaluation	314 (13.3)	1458 (10.9)	701 (16.5)	2189 (12.9)
96152 Health and behavior intervention	197 (8.4)	25 (0.2)	5 (0.1)	0 (0)
99213-99214 Evaluation and management	341 (14.5)	240 (15.2)	315 (7.4)	1060 (6.2)
HCPCS^b^ codes for alcohol, drug therapy	22 (0.9)	1470 (11)	0 (0)	2687 (15.8)
Other	448 (19.1)	4063 (15.1)	177 (4.2)	1346 (7.8)

^a^PHE: public health emergency.

^b^HCPCS: Health Common Procedure Coding System.

**Table 3:** Frequencies and percentages of encounters with each clinician type for the 2 treatment cohorts by time period. Categories with a low n have been combined into the “Other” category.

Clinician type	Telehealth cohort		In-person cohort	
	Pre-PHE^a^, n (%)	PHE, n (%)	Pre-PHE, n (%)	PHE, n (%)
Clinical social worker	783 (32)	2913 (21.8)	3649 (85.9)	9313 (54.7)
Licensed or provisionally licensed counselor	508 (21.6)	3674 (27.4)	0 (0)	1881 (11)
Clinical psychologist	293 (12.5)	2040 (15.2)	88 (2.1)	1797 (10.5)
Psychiatrist and other physician	294 (12.5)	1815 (13.6)	299 (7)	1477 (8.7)
Psychiatric and mental health advanced practice provider	416 (17.7)	1209 (9)	213 (5)	829 (4.9)
Other	72 (3.1)	250 (1.9)	0 (0)	21 (0.1)
Unknown	16 (0.7)	1486 (11.1)	0 (0)	1729 (10.1)

^a^PHE: public health emergency.

## Discussion

### Overview

Behavioral health concerns rose rapidly once the COVID-19 pandemic occurred [7-9]. How behavioral health interventions adjusted to meet this rising need is important to understand. The current pooled data across behavioral health services in 95 rural communities identified multiple changes from the pre-PHE time period to the PHE time period for both the telehealth and in-person cohorts, and these changes included both patient characteristics and treatment processes. In particular, from the pre-PHE period to the PHE period, the telehealth cohort encounters involving anxiety, dissociative, and stress-related disorders increased from 30% (698/2352) to 35%(4632/12,853), and in-person cohort encounters involving substance use disorders increased from 11% (468/4249) to 18% (3048/17,047). Associated with these changes, from the pre-PHE period to the PHE period, telehealth cohort encounters involving treatment service codes for alcohol, drug, and medication-assisted therapy increased from 1% (22/2352) to 11% (1470/13,387), and the in-person cohort increased from 0% (0/4249) to 16% (2687/17,047). The changes observed from the pre-PHE to the PHE period were likely influenced by an interaction of patient, provider, setting, and intervention factors in these nonrandomized cohorts as usual-care approaches adjusted to the COVID-19 pandemic.

A large component of the differences between time periods was due to differences in the patient diagnosis recorded at each encounter. These increases in anxiety, dissociative, stress-related disorders, and substance use disorders are consistent with those found in population studies during the COVID-19 pandemic [16] and have been attributed to pandemic stressors impacting communities as a whole [16,17], as well as the potential psychiatric impacts associated with COVID-19 illness [18,19].

In responding to these changes in patient diagnostic category across time periods, the data indicate that behavioral health clinicians adjusted the treatment service type (in the form of CPT or HCPCS codes) for encounters within both treatment cohorts. Within both the telehealth and in-person cohorts, the percent of encounters involving HCPCS codes for alcohol, drug, and medication-assisted therapy increased substantially. Another change in service that was noted from the pre-PHE to the PHE period was that the telehealth cohort encounters involving 60-minute psychotherapy increased from 8% (190/2352) to 14% (1802/13,387), while in-person cohort encounters involving group therapy decreased from 12% (502/4249) to 4% (739/17,047). The decrease in group therapy is not surprising given that the PHE prompted providers and patients to consider treatment options that met the requirements for social distancing [2,21]. Previous studies have reported that a majority of mental health practitioners reported at least one practice adjustment during the PHE, such as providing additional therapeutic services [22] and that both the process and content of therapy often changed [23]. Changes in the service type likely resulted from a mix of other factors, including patient availability and preferences, changed provider preferences, and changed provider schedules or demands during the pandemic [24,25].

Analyses indicated that the provider type differed between time periods within the 2 treatment cohorts. Within both cohorts, the percent of encounters involving licensed or provisionally licensed professional counselors, clinical psychologists, and clinicians of unknown type increased from the pre-PHE period to the PHE period. The changes in clinician type providing services during the PHE may have resulted from changes by the Centers for Medicare and Medicaid Services and provisions of the US Coronavirus Aid, Relief, and Economic Security Act emergency policies that included authorization for multiple types of clinicians to offer telehealth services, allowance for clinicians to serve out-of-state patients, and improved provider payments for telehealth [1]. Another factor that may have been involved was the need for health systems to adapt to increased patient demand while their workforce needed to adjust to a changing work environment, including more providers delivering telehealth from their home-based rather than office-based settings [26-28].

Previous COVID-19 studies have described how various health systems and clinics-initiated telehealth services for the first time. But the literature on the pandemic has lacked information on the changes in existing behavioral health services regarding adjustments to provider type and services to meet the increased demand and changing patient needs triggered by the pandemic. These findings help to fill that gap by describing how experienced telehealth grantees adjusted their existing services to meet the changing needs presented by the pandemic. How service providers responded to this emergency situation and changing patient diagnoses involved adjustments in the types of clinicians providing treatment and the types of therapy services provided. We hypothesized that patients would show increases in certain psychiatric conditions during the pandemic, and the findings supported this hypothesis. We also hypothesized that treatment types by behavioral health providers would have adjusted accordingly to meet the needs of the changing patient population, and analyses supported that hypothesis along with describing changes in clinician type. To our knowledge, these changes for both telehealth and in-person behavioral health services have not been previously reported. In addition, much of the literature on pandemic changes focuses on urban settings, and this analysis contributes to the sparse literature on rural behavioral health care.

Looking forward, the conclusion of the PHE will provide an opportunity to examine changes as regulations and policies affecting behavioral telehealth either remain in place or return to prepandemic status. Understanding patterns in patient and treatment characteristics could benefit from future research that uses a national set of claims data to examine changes related to the pandemic. As data sets become available, Medicare claims data can be used to examine changing patterns in older populations, but employer-based and Medicaid claims data will be important for elucidating changes affecting younger populations. Also, future research is needed to explicate the interaction of patient, provider, setting, and intervention factors that influenced the patterns observed as a result of the COVID-19 pandemic.

### Limitations

Limitations to the study include the use of convenience samples, which may have introduced multiple biases, including treatment selection biases. In addition, the percentages of unknown clinician types increased during the PHE, which raises uncertainty about changes in those characteristics. Likewise, data are not available on organization changes that might have happened before and after the pandemic including possible changes in health plan contracts. Furthermore, external validity may be limited given the focus on rural patient populations with chronic shortages of behavioral health providers and services. Moreover, the data were limited to a diverse group of grantees as opposed to the general population, and the results reflected averaged changes that likely varied among grantees and treatment sites. Factors beyond the pandemic, such as Medicaid expansion in some states [29], potentially impacted changes in coverage and use. When examining changes from pre-PHE to PHE time periods, it will be important to further differentiate these policy-related effects.

### Conclusions

This study pooled data across 17 behavioral health care systems, all of which had established in-person and telehealth services. Comparisons from the pre-PHE period to the PHE period indicated that patient diagnoses, treatment service type, and clinician type all changed in both the telehealth cohort and the in-person cohort.

## Appendix

Multimedia Appendix 1 Patient characteristics.

## Abbreviations

CPT: current procedural terminology
HCPCS: Health Common Procedure Coding System
IRB: institutional review board
PHE: public health emergency
RUCA: rural-urban commuting area
