# Supplementary material for: Telehealth and In-Person Behavioral Health Services in Rural Communities Before and During the COVID-19 Pandemic: Multisite Prospective Cohort Study
Source: JMIR Ment Health. 2023 Sep 18;10:e47047. doi: 10.2196/47047 (PMC10508259; doi:10.2196/47047)
Supplement: Multimedia Appendix 1 [file mental_v10i1e47047_app1.docx]

Differences within the telehealth and in-person cohorts across the two time periods with respect to patient demographic characteristics are shown in Table 1. The age category of the in-person cohort did not differ significantly from the pre-PHE to the PHE period, but did differ significantly for the telehealth cohort (*P*<.001). In particular, the proportion of patients between ages 18 and 34 rose from 21% (199 out of 958) to 32% (1,206 out of 3,822), and the proportion over age 65 fell from 20% (189 out of 958) to 7% (250 out of 3,822). The sex category of the telehealth cohort did not differ significantly from the pre-PHE to the PHE period, but did differ significantly for the in-person cohort (*P*<.001) with the proportion of male patients increasing from 35% (500 out of 1,437) to 38% (1,918 out of 5,020). The insurance category differed significantly from the pre-PHE to the PHE period for both the telehealth cohort (*P*=.003) and the in-person cohort (*P*<.001). The largest difference was for the in-person cohort where enrollment increased among patients with Medicaid or dual Medicare/Medicaid insurance coverage from 17% (238 out of 1,437) to 28% (1,391 out of 5,020) (*P*<.001). Apparent differences in proportions of racial/ethnic groups between the time periods are likely influenced by a relatively high percentage of enrollees of unknown race/ethnicity in the data set.

**Table 1: Patient characteristics for the two treatment cohorts by time period^a^**

| **Patient Characteristics** | | **Telehealth (N=4,780)** | | | | | | | | **In person (N=6,457)** | | | | | | | |
| --- | --- | --- | --- | --- | --- | --- | --- | --- | --- | --- | --- | --- | --- | --- | --- | --- | --- |
|  |  | **Pre-PHE (N=958)** | | | **PHE (N=3,822)** | | | ***P*-value** | | **Pre-PHE (N=1,437)** | | | **PHE (N=5,020)** | | | ***P*-value** | |
|  |  | **N** | **%** | **N** | | **%** |  | | **N** | | **%** | **N** | | **%** |  | |  |
| **Age category** | |  | | | | | | | |  | | | | | | | |
|  | Less than 18 years | 208 | 21.7% | 955 | | 25.0% | <.001 | | 259 | | 18.0% | 933 | | 18.6% | .187 | |  |
|  | 18 to 34 years | 199 | 20.8% | 1,206 | | 31.6% |  |  | 453 | | 31.5% | 1,642 | | 32.7% |  |  |  |
|  | 35 to 64 years | 362 | 37.8% | 1,400 | | 36.6% |  |  | 550 | | 38.3% | 1,933 | | 38.5% |  |  |  |
|  | >= 65 years | 189 | 19.7% | 250 | | 6.5% |  |  | 175 | | 12.2% | 512 | | 10.2% |  |  |  |
|  | Unknown | 0 | 0.0% | 11 | | 0.3% |  |  | 0 | | 0.0% | 0 | | 0.0% |  |  |  |
| **Sex** | |  | | | | | | | |  | | | | | | | |
|  | Female | 586 | 61.2% | 2,369 | | 62.0% | .078 | | 918 | | 63.9% | 3,097 | | 61.7% | <.001 | |  |
|  | Male | 370 | 38.6% | 1,428 | | 37.4% |  |  | 500 | | 34.8% | 1,918 | | 38.2% |  |  |  |
|  | Other or Unknown | 2 | 0.2% | 25 | | 0.7% |  |  | 19 | | 1.3% | 5 | | 0.1% |  |  |  |
| **Race** | |  | | | | | | | |  | | | | | | | |
|  | White | 732 | 76.4% | 3,057 | | 80.0% | .007 | | 1,237 | | 86.1% | 3,989 | | 79.5% | <.001 | |  |
|  | Black | 16 | 1.7% | 48 | | 1.3% |  |  | 25 | | 1.7% | 152 | | 3.0% |  |  |  |
|  | American Indian | 21 | 2.2% | 110 | | 2.9% |  |  | 22 | | 1.5% | 68 | | 1.4% |  |  |  |
|  | Native Hawaiian or Asian or Multiracial^b^ | 9 | 0.9% | 61 | | 1.6% |  |  | 21 | | 1.5% | 93 | | 1.8% |  |  |  |
|  | Unknown | 180 | 18.8% | 546 | | 14.3% |  |  | 132 | | 9.2% | 718 | | 14.3% |  |  |  |
| **Ethnicity** | |  | | | | | | | |  | | | | | | | |
|  | Hispanic | 26 | 2.7% | 355 | | 9.3% | <.001 | | 31 | | 2.2% | 186 | | 3.7% | <.001 | |  |
|  | Not Hispanic | 771 | 80.5% | 2,769 | | 72.5% |  |  | 1,309 | | 91.1% | 3,972 | | 79.1% |  |  |  |
|  | Unknown | 161 | 16.8% | 698 | | 18.3% |  |  | 97 | | 6.8% | 862 | | 17.2% |  |  |  |
| **Insurance status** | |  | | | | | | | |  | | | | | | | |
|  | Medicaid or Dual | 359 | 37.5% | 1,512 | | 39.6% | .003 | | 238 | | 16.6% | 1,391 | | 27.7% | <.001 | |  |
|  | Medicare | 193 | 20.2% | 716 | | 18.7% |  |  | 268 | | 18.7% | 599 | | 11.9% |  |  |  |
|  | Private insurance | 272 | 28.4% | 1,103 | | 28.9% |  |  | 783 | | 54.5% | 2,171 | | 43.3% |  |  |  |
|  | Self-pay or Uninsured^b^ | 42 | 4.4% | 234 | | 6.1% |  |  | 110 | | 7.7% | 304 | | 6.1% |  |  |  |
|  | Other | 32 | 3.3% | 66 | | 1.7% |  |  | 33 | | 2.3% | 102 | | 2.1% |  |  |  |
|  | Unknown | 60 | 6.3% | 191 | | 5.0% |  |  | 5 | | 0.4% | 453 | | 9.0% |  |  |  |

^a^ All p-values are based on chi-squared tests.

^b^ Categories with low N have been combined.
